# Antithrombotic Effects of Five Organic Extracts of Bangladeshi Plants *In Vitro* and Mechanisms in *In Silico* Models

**DOI:** 10.1155/2015/782742

**Published:** 2015-05-17

**Authors:** Sakib Mahmud, Samina Akhter, Md. Atiar Rahman, Jannatul Aklima, Shaheen Akhter, Syeeda Rayhana Merry, S. M. Rakibul Jubair, Raju Dash, Talha Bin Emran

**Affiliations:** ^1^Department of Biochemistry and Molecular Biology, University of Chittagong, Chittagong 4331, Bangladesh; ^2^Bangladesh Forest Research Institute, Chittagong 4000, Bangladesh; ^3^Department of Pharmacy, BGC Trust University, Chittagong 4000, Bangladesh

## Abstract

This research was carried out to investigate the thrombolytic effects of the methanolic extracts of five Bangladeshi plants. Phytochemical metabolites of those plants have been identified to elucidate whether the plant-derived metabolites are linked with the thrombolytic effects. Potential computer aided models were adopted in this study to find out a structure-function correlation between the phytochemical constituents and thrombolytic effects using the secondary metabolites as ligands and tissue plasminogen activator (t-PA) as receptor for the best fit ligand-receptor interaction.

## 1. Introduction

Thrombosis is a fatal disease which is characterized by the formation of blood clots (thrombus) in the circulatory system because of the imbalance of homeostatic system of physiological procedures. This is a critical event in the arterial diseases connected with acute coronary disorders such as pulmonary emboli, deep vein thrombosis, strokes, heart attacks, and venous thromboembolic disorders that account for sudden morbidity and mortality. Thrombosis leads to vascular blockade and while recovering it causes fatal consequences, such as cerebral or myocardial infarction and even death [1].

Thrombolytic agents that include tissue plasminogen activator (t-PA), alteplase, anistreplase, urokinase (UK), and streptokinase (SK) are widely used throughout the world for the treatment of thromboembolic diseases although streptokinase and urokinase are the first choices in Indian regions due to the easy reach and lower cost [2, 3] as compared to other thrombolytic drugs. But the weak substrate specificity of these first-generation drugs (streptokinase and urokinase) commonly leads to some major side effects such as anaphylactic reaction, systemic fibrinolysis, and hemorrhage [4]. Immunogenicity is another important issue which restricts the multiple treatments of a given patient with streptokinase [5]. Because of the setbacks of the available thrombolytic drugs, attempts are underway to develop improved recombinant variants of these drugs [6, 7]. Thrombolytic therapy with recombinant t-PA is effective in acute myocardial infection, but the treatment is limited by a fairly slow reperfusion rate and frequent early reocclusions. Moreover, the platelet-rich thrombi are highly resistant to lysis by t-PA [8]. Due to the limited scopes of almost all the synthetic and recombinant antithrombotic drugs, the mergence for alternatives is highly necessitated. Previously we reported few Bangladeshi plants showing mild to potent thrombolytic effects [9]. This research aims to investigate the thrombolytic effects of some other five Bangladeshi medicinal plants such as* Ocimum tenuiflorum*,* Andrographis paniculata*,* Adhatoda vasica*,* Leea macrophylla*, and* Litsea glutinosa*. These plants have been chosen based on their traditional uses by local communities, herbalists, or traditional healers. Researchers reported their common uses in cardiovascular diseases, atherosclerosis, blood impurities, and other relevant disorders.* Ocimum tenuiflorum* has also been shown to counter metabolic stress through normalization of blood glucose, blood pressure and lipid levels, and psychological stress through positive effects on memory and cognitive function and through its anxiolytic and antidepressant properties [10].* Andrographis paniculata* has long been used in cardiovascular diseases. It is a spontaneous hypotensive agent. Different components of this plant are also involved in heart diseases [11].* Adhatoda vasica* is used as a blood purifier [12].* Leea macrophylla* is reported to be used in effusion of blood [13].* Litsea glutinosa* leaf extract is used in cardiovascular activities [14]. In spite of their uses in relevant disorders, there is little or no scientific evidence to be used as therapeutically proven drugs. This research, therefore, investigated not only their thrombolytic effects but also the probable mechanism, through* in silico* docking model, of how they are involved in such biological action.

## 2. Materials and Methods

### 2.1. Plant Collection and Identification

The plants* Ocimum tenuiflorum*,* Andrographis paniculata*, and* Adhatoda vasica *were cultivated and harvested from Rangamati, Khagrachari, and Bandarban hill tracts area, respectively. They were harvested in the month of July-August (2013) and they were preserved in Bangladesh Forest Research Institute, Chittagong, at low temperature (16–20)°C.* Leea macrophylla *was collected from the cultivated area of Bangladesh Council of Scientific and Industrial Research (BCSIR), Rajshahi, whereas* Litsea glutinosa *leaves were collected from Chittagong University Campus, Bangladesh*. Leea macrophylla *and* Litsea glutinosa *were also preserved at low temperature (16–20)°C in the Laboratory of Phytomedicine, Department of Biochemistry and Molecular Biology, University of Chittagong. The plants were authenticated as* Ocimum tenuiflorum*,* Andrographis paniculata*,* Adhatoda vasica*,* Leea macrophylla*, and* Litsea glutinosa* by Dr. Shaikh Bokhtear Uddin, Taxonomist and Associate Professor, Department of Botany, University of Chittagong.

### 2.2. Chemicals and Reagents

To the commercially available lyophilized streptokinase (SK) vial (Incepta Pharma. Co. Ltd., Dhaka, Bangladesh) of 1500000 I.U., 5 mL sterile distilled water was added and mixed properly. This suspension was used as a stock from which 100 *μ*L (30,000 I.U.) was used for* in vitro* thrombolysis. Hexane (99.5%) and methanol (99%) were purchased from the local sources and they were of reagent grade.

### 2.3. Preparation of Extracts

Plant materials were washed properly and chopped into small pieces to make a semished sun-dry for seven days. After drying, plant materials were powdered with mechanical grinder (Willey mill). The resulting powder was defatted through hexane followed by soaking into methanol for eight days with an occasional stirring while the filtrate was collected after every two days using cheesecloth and Whatman filter paper number 1. The filtrate was concentrated under reduced pressure at the temperature below 50°C using rotatory evaporator (RE 200, Bibby Sterling Ltd., UK) to find crude extract in glass Petri dishes (90 × 15 mm, Pyrex, Germany). The crude was allowed to dry for the complete evaporation of solvent at 37°C [15]. The crude methanol fractionation was obtained using protocol designed by Kupchan et al. [16]. These concentrated extracts were used to investigate antithrombotic effect of the mentioned medicinal plants.

### 2.4. Sample Preparation and Qualitative Phytochemical Screening

The crude methanol extracts of* Ocimum tenuiflorum*,* Andrographis paniculata*,* Adhatoda vasica*,* Leea macrophylla*, and* Litsea glutinosa* were undertaken for phytochemical screening in order to detect the presence (or absence) of alkaloids, flavonoids, steroids, tannins, saponins, phlobatannins, and glycosides. 100 mg of each of the extracts was suspended in 10 mL distilled water and the suspension was shaken vigorously on a vortex mixer. The suspension was kept overnight and decanted to remove the soluble supernatant, which was filtered through a 0.22 *μ*m syringe filter. 100 *μ*L of this aqueous preparation was added to the micro centrifuge tubes containing the clots to check thrombolytic activity. This experimental approach was carried out using standard techniques as described below [17–19].

#### 2.4.1. Test for Alkaloids

5 mg of extract was taken in a test-tube. 2.0 mL of 1% HCl is added to the test-tube. Mixture was stirred on a steam bath. After that, cooling and filtering were done. A few drops of Mayer's reagent were added to it. A creamy precipitation indicates the presence of alkaloids.

#### 2.4.2. Test for Flavonoids (NaOH Test)

1 mL of extract of each plant species was taken in test-tubes. A few drops of 1% HCl were added to the test-tubes. Then 2 mL of NaOH was added. A canary yellow color indicates the presence of flavonoids.

#### 2.4.3. Test for Steroids (Salkowski's Test)

1 mL of each plant extract was taken in test-tubes. 1 mL of concentrated sulfuric acid (H_2_SO_4_) was added to the test-tubes. Appearance of a clear reddish brown color ring at the interface confirms the presence of steroids.

#### 2.4.4. Test for Tannins

1 mL of each plant extract was taken in test-tubes. Two drops of freshly prepared FeCl_3_ were added to it. Dark black color precipitation can be observed which gives green black to blue black color on dilution which indicates the presence of tannins.

#### 2.4.5. Test for Saponins

1 mL of each plant extract was taken in test-tubes. 2.5 mL of distilled H_2_O is added to the test-tubes. Test-tubes with solution were vigorously shaken and allowed to stand for few minutes at room temperature. A persistent frothing indicates the positive results for saponins.

#### 2.4.6. Test for Phlobatannins

1 mL of each plant extract was taken in test-tubes. 2.5 mL of 2% HCl was added to it. Formation of precipitation indicates the positive results for phlobatannins.

#### 2.4.7. Test for Cardiac Glycosides

2 mL of each plant extract was taken in test-tubes. 1 mL of glacial acetic acid was added. After that, 1 drop of liquid FeCl_3_ was added and then 0.5 mL of concentrated H_2_SO_4_ was added to the test-tube. Brown ring at the interface indicates the presence of glycosides.

### 2.5. Antithrombotic Effects

#### 2.5.1. Blood Specimen

From twenty healthy human volunteers, six milliliters of the whole blood was drawn without a history of oral contraceptive or anticoagulant therapy using a protocol approved by the Institutional Ethics Committee of Chittagong University, Faculty of Medicine. An earlier consent, approval number HET-CU2013/3, was taken from the Faculty of Medicine, University of Chittagong, for collection of blood samples from human volunteers. 500 *μ*L of blood was transferred to each of the seven previously weighed micro centrifuge tubes to form clots.

#### 2.5.2. Consent of Informed Donor

A consent form mentioning research project title, name, and details of investigators contacts as well as the purpose of the research was supplied to the volunteer donors. They were also supplied with the detailed description of the inclusion and exclusion criteria of the donors, whether donors will receive any therapy or not, volume of blood to be taken, possible discomfort of the puncture sites, and time required for blood sampling. Explanation was made on if future use of the research data beyond the current study is anticipated, whether this is a focus group if so the Principal Investigator should put a procedure in place in which the researchers caution people against the limit on confidentiality. Access to research information regarding who would have access to the collected sample and information regarding retention of sample and schedules for their disposal was also detailed. It was indicated in the consent form that the volunteers might refuse to donate blood at any time. Donor whether could withdraw his sample data was disclosed. The sample that was restricted for that individual study not for future research projects was presented in the consent form. Possible complications, for example, the possibility of bruising or swelling while giving blood, or some other discomforts at the site where blood is drawn, and that there might be minimal chance of infection and that these discomforts were brief and transient, were also informed. The potential benefits of this study, not directly of the donors but the society in general or individuals with a similar condition that might be benefitted from the results of the study, were explained. Confidentiality statement was included in the consent form in the way that “confidentiality will be respected and no information that discloses the identity of the participant will be released or published without consent unless required by law of states. The legal obligation includes a number of circumstances, such as suspected child abuse and infectious disease, expression of suicidal ideas where research documents are ordered to be produced by a court of law and where researchers are obliged to report to the appropriate authorities. In those rare instances where it will not be possible to assure complete confidentiality,” the limits on this obligation were carefully explained. The signatures with date of the donors were also included in the consent form.

#### 2.5.3. Determination of Clot Lysis

Clot lysis approaches were carried out as reported earlier [20]. 6 mL venous blood drawn from the healthy volunteers was distributed in 10 different preweighed sterile micro centrifuge tubes (0.5 mL/tube) and incubated at 37°C for 45 min. After clot formation, serum was completely removed without disturbing the clot and each tube having clot was again weighed to determine the clot weight (clot weight = weight of clot containing tube − weight of tube alone). 100 *μ*L of methanol extracts of* Ocimum tenuiflorum*,* Andrographis paniculata*,* Adhatoda vasica*,* Leea macrophylla*, and* Litsea glutinosa* was added separately. As positive and negative controls, 100 *μ*L of SK and distilled water, respectively, was added separately. All the tubes were then incubated at 37°C for 90 min and observed for clot lysis. Released fluid was removed and tubes were again weighed to observe the difference in weight after clot disruption. Difference obtained in weight taken before and after clot lysis was expressed as percentage of clot lysis. The experiment was repeated with the blood samples of the informed donors.

#### 2.5.4. Molecular Docking Analysis for Thrombolytic Mechanism

For molecular docking studies, the data from databases used in this study include PDB (Protein Data Bank) [21] and Pubchem [22]. For docking analysis, the protein file was prepared through the receptor preparing wizard in FlexX [23] which includes Flexx (LeadIT 2.1.6). FlexX, a fully automated docking program available on LeadIT 2.1.6 package, was used to dock compound into the active site of the enzymes. FlexX is a fragment based docking algorithm, which builds putative poses of the ligands using an incremental construction approach. FlexX considers ligand flexibility by changing the conformations of the ligand in the active site while making the protein rigid [23].

*(1) Docking with FlexX. *FlexX (which is now a part of LeadIT) is a flexible docking method that uses an incremental construction (IC) algorithm and a pure empirical scoring function similar to the one developed by Böhm and coworkers to place ligands into the active site [24]. IC algorithms first dissect each molecule into a set of rigid fragments according to rotatable bonds and then incrementally assemble the fragments around the binding pocket [23]. For docking studies, a receptor description file was prepared through the FlexX graphic interface. An active site was defined by selecting the residue of the protein. The active site includes protein residues around 10 Å radius sphere centered on the center of mass of the ligand. Based on energy values, top ten ranked poses for each ligand in data set were selected for further analysis.

The free binding energy Δ*G* of the protein-ligand complex is given by

(1) ΔG=ΔG0+ΔGrot⁡×Nrot⁡+ΔGhb∑neutral H bondsfΔR,Δα+ΔGio∑ionic int.fΔR,Δα+ΔGar∑aro int.fΔR,Δα+Glipo∑lip ocont.f∗ΔR.

Here, *f*(Δ*R*, Δ*α*) is a scaling function penalizing deviations from the ideal geometry and *N*_*rot*⁡_ is the number of free rotatable bonds that are immobilized in the complex. The terms Δ*G*_hb_, Δ*G*_io_, Δ*G*_ar_, and Δ*G*_0_ are adjustable parameters. Δ*G*_lipo_ is lipophilic contact energy [23, 25].

### 2.6. Statistical Analysis

The calculated significance between the percentages of clot lysis by SK and plant extracts of* Ocimum tenuiflorum*,* Andrographis paniculata*,* Adhatoda vasica*,* Leea macrophylla*, and* Litsea glutinosa* was tested by the paired *t*-test analysis using the software SPSS, version 18.0 (SPSS for Windows, Version 18.0, IBM Corporation, New York, USA). Data are expressed as mean ± SD. The mean difference between positive and negative controls was considered significant at *P* values that were less than 0.05.

## 3. Results and Discussion

### 3.1. Results

Physical properties and yields of the crude extracts are summarized in Table 1. Qualitative phytochemical screening of the extracts revealed the presence of different secondary metabolites (Table 2). The presence of flavonoids, steroids, and cardiac glycosides was consistently noted in all the plant extracts. Alkaloids were present in* Adhatoda vasica* and* Litsea glutinosa* but absent in others. Only* Leea macrophylla* showed the phlobatannins. Tannins and saponins were absent in* Leea macrophylla*. Tannins were not present in* Andrographis paniculata.*

**Figure 1:**
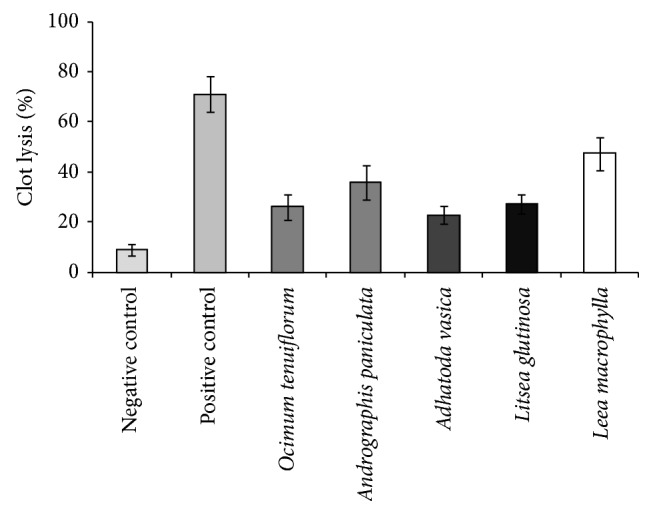
Comparative clot lysis by streptokinase, water, and methanol extract of* Ocimum tenuiflorum*,* Andrographis paniculata*,* Adhatoda vasica*,* Leea macrophylla*, and* Litsea glutinosa*.

In antithrombotic approach with human blood sample, addition of 100 *μ*L streptokinase, a positive control (30,000 I.U.), to the clots and subsequent incubation for 90 minutes at 37°C showed 71.14 ± 6.91% clot lysis. On the other hand, distilled water was treated as negative control which showed only 8.89 ± 2.22%, a negligible clot lysis. The mean difference in clot lysis percentage between positive and negative controls was very significant (*P* values less than 0.001).* Leea macrophylla* showed the highest significant (47.47 ± 6.65%) clot lysis activity among all the extracts (*P* values < 0.001).* Andrographis paniculata* showed 35.74 ± 6.76% of clot lysis and its *P* value was less than 0.001.* Ocimum tenuiflorum *and* Litsea glutinosa* showed 26.08 ± 5.12% and 27.25 ± 3.97% of clot lysis, respectively.* Adhatoda vasica* showed 22.86 ± 3.61% clot lysis and the value was very significant (*P* values < 0.001). Percentages of clot lysis obtained after treating the clots with different organic extracts and appropriate controls are shown in Table 3 and their comparison was represented in Figure 1.

**Figure 2:**
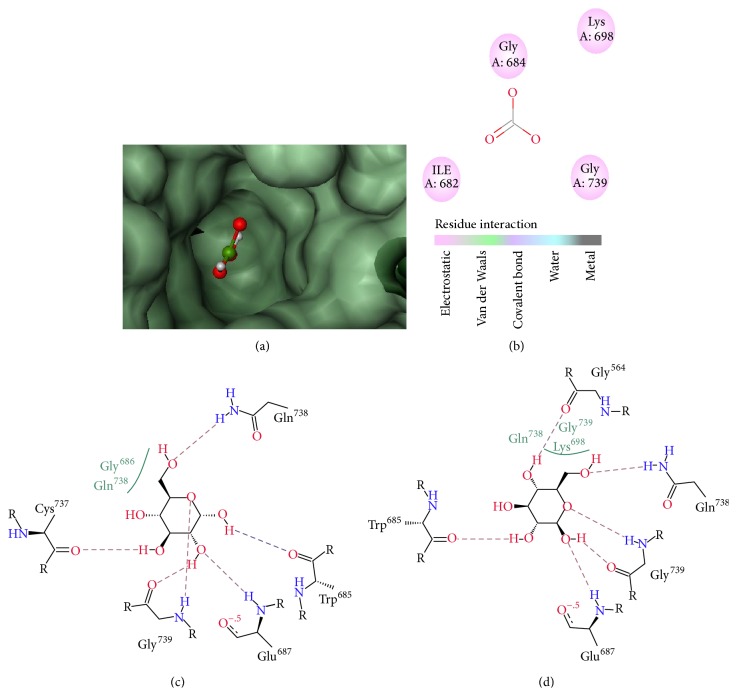
(a) Receptor cavity of reference ligand of tissue plasminogen (Pg, pdb id: 4DUR), (b) active site residues of SK binding site. Interaction of (c) *α*-d-glucopyranose and (d) *β*-d-glucopyranose with the tissue plasminogen protein.

Presence of three common and major metabolites in all the experimental extracts was considered as the basis to undertake the metabolites for molecular docking analysis. Molecular docking is an effective and fast computational technique to estimate the binding affinity of a ligand (drug candidate) in the macromolecular target site (receptor). The active site was identified and considered the reference ligand binding in the position of Lys-698 residues (SK binding site) shown in Figures 2(a) and 2(b). However, after docking simulation done by flexX simulation, it was found that only *α*-d-glucopyranose (CID79029 and CID64689) and *β*-d-glucopyranose considered as glycoside skeleton showed the maximal binding energies which were −14 kj/mol and −15 kj/mol, respectively. The postdocking analysis suggests that *α*-d-glucopyranose was involved in the formation of six salt bridges with Cys^737^, Gly^739^, Glu^687^, Trp^685^, and Gln^738^ (Figure 2(c)) and other residues such as Gly^686^. In case of *β*-d-glucopyranose (Figure 2(d)), six hydrogen bonds with Gly^564^, Gln^738^, Trp^685^, Gly^739^, Glu^687^, and Lys^698^ residues were formed. However, Lys^698^ was found to act as a contributor of hydrophobic bond. Ligand efficiency of *α*-d-glucopyranose was 0.27 and that of *β*-d-glucopyranose was 0.30. No binding efficiency was observed for the basic skeleton of flavonoids and steroids.

### 3.2. Discussion

Advances in phytochemistry and identification of plant compounds, which are effective in curing certain diseases, have renewed the interest in herbal medicines. About 30% of the pharmaceuticals are prepared from plants worldwide [26, 27]. Phytochemical analysis conducted on the plant extracts revealed the presence of constituents which are known to exhibit medicinal as well as physiological activities [17]. In this research, all the experimented plant extracts exhibited flavonoids, steroids, and cardiac glycosides. The activities of flavonoids are due to their ability to form complex with extracellular and soluble proteins [28]. They are also effective as antioxidant and antiplatelet [29–33]. Glycosides are known to lower the blood pressure according to many reports [34].

A number of studies have been conducted by various researchers to find out the herbs and natural food sources and their supplements having antithrombotic (anticoagulant and antiplatelet) effect and there is evidence that consuming such food leads to prevention of coronary events and stroke [35–37]. Herbal preparations, if taken in appropriate dose, can lead to a better option for curing various ailments. In our thrombolytic assay, the comparison of positive control with negative control clearly demonstrated that clot dissolution does not occur when water was added to the clot. When compared with the clot lysis percentage obtained through SK and water, a significant (*P* < 0.05) thrombolytic activity was observed after treating the clots with the extracts. Methanol extract of* Leea macrophylla* showed the highest and* Adhatoda vasica *showed the lowest thrombolytic effects.* Andrographis paniculata* showed thrombolytic effects close to those of* Leea macrophylla *although the extent of glycosides in* Andrographis paniculata* is much lower than that of the latter one. The phenomenon could be explained as a fact that the active constituent in both the glycosides might be different while the active glycosidic ingredient of* Andrographis paniculata* could be manifolds stronger than that of* Leea macrophylla* suggesting that not only the extent of an individual type of metabolite but also the active ingredient of that sort of metabolite is also important for biological activity. And we investigated the type and extent of secondary metabolites in our research but not the active principle in the extract.

As discussed earlier, all the experimental plant extracts exhibited flavonoids, steroids, and glycosides. So, it is our interest to know which secondary metabolite is particularly involved in the activation of tissue plasminogen activator because as we know, tissue plasminogen activator (t-PA) is a serine protease that converts plasminogen (Pg) to plasmin and can trigger the degradation of extracellular matrix proteins or clots and thus exerts thrombolysis [38, 39]. It is that the standard drug streptokinase (SK), a three-domain protein, is involved in the activation of tissue plasminogen and forms a tight stoichiometric complex with Pg, changing the zymogen proteolytic specificity from fibrin to the activation of other Pg molecules [40]. It was anticipated that Lys-698 (156) in human Pg plays a key role in the contact activation mechanism [41, 42]. This lysine residue is located in the activation pocket of Pg shielded from bulk solvent by the flexible autolysis loop. Molecular modeling suggests that it is feasible for the lysine side chain to reach a position from which it forms a salt bridge bond with Asp-740 (194) under the influence of the cofactor SK, thus triggering active site formation during contact activation [41]. Regarding this, we undertook the general skeleton of flavonol, steroid, and cardiac glycoside to find out their interaction on the SK binding site of Pg which was done by molecular docking simulation.

Molecular docking is an effective and fast computational technique to estimate the binding affinity of a ligand (drug candidate) in the macromolecular target site (receptor). A scoring system is used to detect the ideal docking configuration. Scoring systems usually use entropy maximization strategies, which generally depend on electrostatic attraction forces, Van der Waal's forces, and hydrophobic interactions [43]. In this scoring system, the crystal structure of tissue plasminogen was downloaded from protein data bank (pdb id: 4DUR) and the 3D structure of general skeleton of flavonol, glycoside (*α*-d-glucopyranose and *β*-d-glucopyranose), and steroid was obtained from Pubchem databases. The protein file was prepared through the receptor preparing wizard in FlexX [23]. The active site was identified and Lys-698 residues (Sk binding site) were considered as the reference ligand binding [44]. However, after docking analysis by flexX simulation through incremental search, only alpha-d-glucopyranose (CID79029 and CID64689) and beta-d-glucopyranose were found to show the maximal free binding energies, −14 kj/mol and −15 kj/mol, respectively. A positive value binding energy for flavonoids was recorded while no docking confirmation for steroid was noted. The postdocking analysis suggests that alpha-d-glucopyranose was involved in the formation of six salt bridges with Cys^737^, Gly^739^, Glu^687^, Trp^685^, Gln^738^, and Gly^686^. Out of these six salt bridges, Gly^686^ was involved in hydrophobic interaction and it possesses moderate ligand efficiency 0.27. In contrast, the ligand protein complex for beta-d-glucopyranose formed six hydrogen bonds with Gly^564^, Gln^738^, Trp^685^, Gly^739^, and Glu^687^ residues. However, Lys^698^, in that case, was found to act as a contributor of hydrophobic bond. And ligand efficiency of beta-d-glucopyranose was 0.30. The docking simulation study, on the basis of the above result, suggested that only glycoside is responsible for thrombolytic mechanism as it has the interaction on the activation site of tissue plasminogen activator that converts plasminogen to plasmin and can trigger the degradation of extracellular matrix proteins or clots and thus exerts thrombolysis.

## 4. Conclusion

Phytochemical screening revealed the presence of flavonoids, steroids, and glycosides in all the extracts. From this study, thrombolytic activity of* Leea macrophylla* and* Andrographis paniculata *methanol extracts has been found to show promising* in vitro* clot lysis activity whereas* Ocimum tenuiflorum*,* Adhatoda vasica*, and* Litsea glutinosa *were found to show moderate-to-mild thrombolytic activity. Molecular docking analysis suggested the glycosides of these plants to be the major molecules that contribute to the observed thrombolytic/antithrombotic effects. However, other metabolites might have the contribution in these effects but that is not revealed at least in our experimental design.

## Figures and Tables

**Table 1:** Methanol extracts of *Ocimum tenuiflorum*, *Andrographis paniculata*, *Adhatoda vasica*, *Leea macrophylla*, and *Litsea glutinosa* and their physical properties.

Plant name	Solvent	Powder (g)	Crude extract (g)	Yield (%)	Crude physical appearance
*Ocimum tenuiflorum *	Methanol	320	25.67	8.02	Deep green with presence of arbitrary shaped crystals
*Andrographis paniculata *	Methanol	880	83.34	9.47	Deep green gummy mass
*Adhatoda vasica *	Methanol	921	31.97	3.47	Green with reddish gummy mass
*Leea macrophylla *	Methanol	600	33.6	5.6	Greenish semisolid
*Litsea glutinosa *	Methanol	450	20	4.4	Black

**Table 2:** Qualitative phytochemical screening of *Ocimum tenuiflorum*, *Andrographis paniculata*, *Adhatoda vasica*, *Leea macrophylla*, and *Litsea glutinosa*.

Chemical constituent	Name of the species				
	*Ocimum tenuiflorum *	*Andrographis paniculata *	*Adhatoda vasica *	*Leea macrophylla *	*Litsea glutinosa *
Alkaloids	—	—	+	—	++
Flavonoids	+++	++	+	++	++
Steroids	+++	++	++	++	++
Tannins	+++	-	++	—	++
Saponins	++	+++	+	—	++
Phlobatannins	—	—	—	+	—
Glycosides	++	+	+	+	++

Note: “+,” “++,” and “+++” indicate the mild, moderate, and strong presence, whereas “—” indicates the absence of secondary metabolites in respective extract.

**Table 3:** *In vitro* clot lysis activity of *Ocimum tenuiflorum*, *Andrographis paniculata*, *Adhatoda vasica*, *Leea macrophylla*, and *Litsea glutinosa*.

Herbs/drugs	Fractions	% of clot lysis (mean ± SD)	*P* value (two-tailed) when compared to negative control (water)
Negative control (water)	Methanol	8.89 ± 2.22	—
Positive control (streptokinase)	Methanol	71.14 ± 6.91	*P* < 0.001
*Ocimum tenuiflorum *	Methanol	26.08 ± 5.12	*P* < 0.001
*Andrographis paniculata *	Methanol	35.74 ± 6.76	*P* < 0.001
*Adhatoda vasica *	Methanol	22.86 ± 3.61	*P* < 0.001
*Leea macrophylla *	Methanol	47.47 ± 6.65	*P* < 0.05
*Litsea glutinosa *	Methanol	27.25 ± 3.97	*P* < 0.001

Values are mean ± SD (*n* = 20); ^*^ *P* < 0.05, ^**^ *P* < 0.001, Dunnett test as compared to control (positive and negative). Statistical representation of the effective clot lysis percentage by herbal preparations, positive thrombolytic control (streptokinase), and negative control (sterile distilled water) processed by paired *t*-test analysis (Dunnett test).
